# The Role of RFRP Neurons in the Allostatic Control of Reproductive Function

**DOI:** 10.3390/ijms242115851

**Published:** 2023-11-01

**Authors:** Maggie C. Evans, Greg M. Anderson

**Affiliations:** Centre for Neuroendocrinology and Department of Anatomy, School of Biomedical Sciences, University of Otago, Dunedin 9016, New Zealand; maggie.c.evans@gmail.com

**Keywords:** RFRP neurons, RFRP-3, reproduction, stress, puberty, seasonal breeding

## Abstract

Reproductive function is critical for species survival; however, it is energetically costly and physically demanding. Reproductive suppression is therefore a physiologically appropriate adaptation to certain ecological, environmental, and/or temporal conditions. This ‘allostatic’ suppression of fertility enables individuals to accommodate unfavorable reproductive circumstances and safeguard survival. The mechanisms underpinning this reproductive suppression are complex, yet culminate with the reduced secretion of gonadotropin-releasing hormone (GnRH) from the hypothalamus, which in turn suppresses gonadotropin release from the pituitary, thereby impairing gonadal function. The focus of this review will be on the role of RFamide-related peptide (RFRP) neurons in different examples of allostatic reproductive suppression. RFRP neurons release the RFRP-3 peptide, which negatively regulates GnRH neurons and thus appears to act as a ‘brake’ on the neuroendocrine reproductive axis. In a multitude of predictable (e.g., pre-puberty, reproductive senescence, and seasonal or lactational reproductive quiescence) and unpredictable (e.g., metabolic, immune and/or psychosocial stress) situations in which GnRH secretion is suppressed, the RFRP neurons have been suggested to act as modulators. This review examines evidence for and against these roles.

## 1. Introduction

Reproductive function is fundamental for species survival. However, it is physiologically costly—especially in mammals—due to the high energetic demands of pregnancy, lactation and rearing of offspring. When environmental or physiological conditions are not conducive for reproduction, neuroendocrine pathways that can exert allostatic control over reproductive function cause acute reproductive suppression. This ‘allostatic’ reproductive suppression is adaptive, as it enables individuals to accommodate unfavorable changes in their environment and/or in their physiology in order to safeguard survival. According to McEwen and Wingfield [1], allostasis is “achieving stability through change”—a fundamental process through which an individual actively adjusts its physiology and/or behavior to accommodate both predictable and unpredictable events.

There are many examples of allostatic reproductive suppression. For example, in seasonal breeders, reproductive suppression is predictable, such that fertility is coordinated to ensure offspring are born when environmental conditions are most favorable for survival. During different life cycle stages, such as pre-puberty and post-menopause, reproductive function is also predictably suppressed. However, unpredictable events can also cause reproductive suppression, such as certain environmental and/or physiological states. During times of severe metabolic stress, for example due to famine, energy demand exceeds energy supply, which will initiate an allostatic response that directs an individual away from energetically costly breeding and into survival mode. During times of psychosocial trauma or severe illness, which are also unfavorable times to reproduce, sufficient energy may be present, but the activation of the stress systems can induce a facultative physiological and/or behavioral response that ultimately defers reproduction [1,2]. Allostatic reproductive suppression encompasses all forms of reproductive suppression that represent an accommodation or adaptation that ultimately serves to safeguard the organism’s survival.

To understand the mechanisms underpinning allostatic reproductive suppression, it is necessary to first understand how the reproductive axis—the hypothalamo-pituitary-gonadal (HPG) axis—is regulated. The hypothalamic gonadotropin-releasing hormone (GnRH) neurons governing the HPG axis serve as the final integrators of a multitude of neuroendocrine and hormonal inputs [2]. GnRH release stimulates pituitary gonadotropin secretion (luteinizing hormone, LH, and follicle-stimulating hormone, FSH) either as frequent pulses or a large pre-ovulatory surge, which in turn drive gonadal function. Gonadal steroids exert feedback modulation of the axis at both the hypothalamic and pituitary levels [3,4]. Allostatic reproductive suppression is generally coordinated through neuroendocrine mechanisms that culminate in reduced GnRH release, thereby causing reduced LH/FSH release and suppressed gonadal function. The network of afferent inputs modulating GnRH neurons are collectively referred to as the ‘GnRH neuronal network’, and include inputs relaying information ranging from nutritional and immune status cues to circadian and temperature cues, to psychosocial and environmental stress cues. The multitude of neuroendocrine pathways converging on the GnRH neuronal network is vastly complex. However, the focus of this review will be on a particular neuronal population that appears to play a prominent role in mediating the allostatic suppression of fertility—the RF amide-related peptide (RFRP)-producing neurons.

## 2. RFRP-3 in Reproductive Axis Control

RFamides form a class of neuropeptides characterized by an arginine–phenylalanine–amide motif at their C terminus. They were first identified in 1977 in molluscan cerebral ganglia, but almost another decade passed before they were purified and chemically characterized in any mammalian species. There are now five RFamide families known to exist in the mammalian central nervous system, where their widespread fiber distribution implies varied roles including regulation of energy balance, behavior, and reproduction [5]. The RFRP neuronal population was discovered in 2000 [6] with cell bodies scattered in and around the dorsomedial hypothalamus (DMH) that secretes RFRP-1 and RFRP-3 peptides. Of these, RFRP-3 (also referred to as neuropeptide VF in mammals) [7] has received by far the most attention with regard to the modulation of reproductive function. It is mostly inhibitory to GnRH activity and LH secretion [8,9], hence the name usually applied to its avian ortholog—gonadotropin-inhibitory hormone (GnIH) [10]. Limited evidence suggests RFRP-3 fibers extend into the external layer of the median eminence of some mammalian species, including humans, which implies the potential for the direct regulation of gonadotropin release [11]. Similarly, there is somewhat limited in vivo evidence to support the actions of RFRP-3 directly on pituitary gonadotrophin secretion in mammals. There is little, if any, consistency in the reported effects of peripherally administered RFRP-3 on LH secretion [8,12,13,14,15,16]. For example, in postmenopausal women, peripherally administered RFRP-3 suppressed LH secretion, whereas in males, who were treated with kisspeptin-10 to elevate LH secretion, co-administration with RFRP-3 did not reduce kisspeptin-stimulated LH secretion [17]. RFRP-3 concentrations in the portal blood supplying the anterior pituitary gland do not correlate with LH pulses [18] or stress treatments [19] in ewes, and most (but not all [11]) reports of RFRP immunoreactive fibers innervating the external zone of the hypothalamic median eminence—the ‘neurosecretory zone’—are negative; see [5] for review. Evidence for hypothalamic actions of RFRP-3 on the GnRH neurons is more consistent; however, there are obviously no functional data available on this for humans. The firing rate of a subpopulation of GnRH neurons in mouse brain slice preparations has been shown to be inhibited by RFRP-3 [20,21], and a comparable subpopulation expresses the RFRP-3 receptor NPFFR1 (also known as GPR147) [16,22,23] in mice. The focus of this review will therefore be on the central effects of RFRP-3 on GnRH neurons. One key event of the female reproductive cycle that may be modulated by a *decrease* in RFRP neuronal activity is the preovulatory GnRH/LH surge [24]. Hypothalamic *Rfrp* gene expression, RFRP cell numbers, and RFRP + cFos coexpression (indicating RFRP neuronal activation) all decline on the afternoon of the surge in naturally cycling or surge-induced hamsters and mice [25,26]. This decrease in RFRP-3 signaling might lead to the disinhibition of GnRH neurons, thereby augmenting the surge (which is known to be primarily driven by stimulatory kisspeptin inputs to GnRH neurons) [2,3,4].

**Figure 1:**
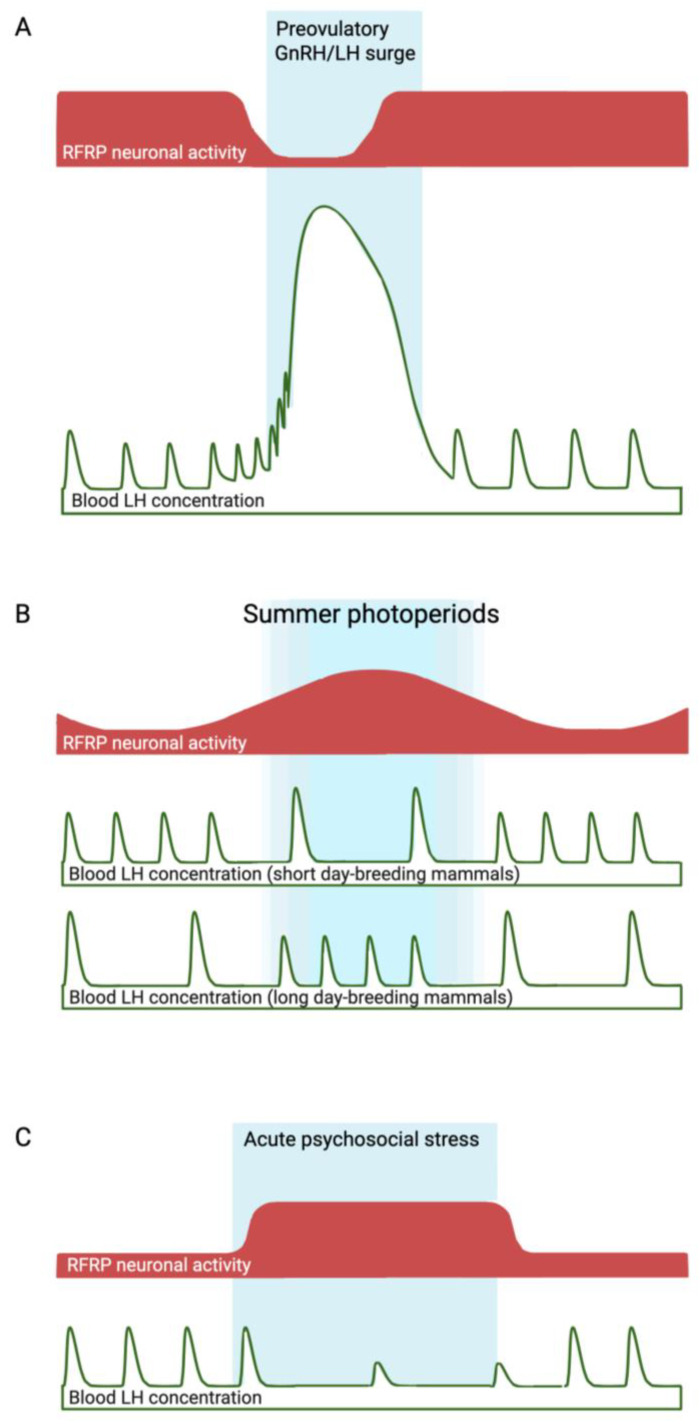
Fluctuations in RFRP neuronal activity (red shaded areas) that are hypothesized to contribute to the preovulatory GnRH/LH surge (**A**), and seasonal (**B**) or psychosocial stress-induced (**C**) infertility (delineated by blue shaded areas). In the case of seasonal infertility (a predictable time of reproductive suppression), the period of heightened inhibitory RFRP neuronal activity during long summer photoperiods correlates with the seasonal anestrous period of ‘short-day breeders’, but not with that of small mammals, which become reproductively active in response to long photoperiods. The evidence linking heightened inhibitory RFRP neuronal activity during psychosocial stress (an unpredictable cause of reproductive suppression) with reduced LH pulsatility is stronger, since experimental silencing of RFRP neurons has been shown to overcome the effects of stress. In the examples described in the text, RFRP neuronal activity has been estimated at a limited number of time points based on *Rfrp* mRNA levels, RFRP immunoreactivity, and cFos expression in RFRP neurons. Continuous measures of neuronal activity, such as in vivo fiber photometry, should provide more comprehensive supporting evidence linking RFRP neuronal fluctuations to changes in reproductive activity in these and other situations.

As mentioned previously, RFRP neurons appear to be involved in both the temporal regulation of reproductive function, which causes predictable cycles of reproductive activation and inactivation, as well as in stress-related reproductive regulation, which allows for facultative reproductive suppression. RFRP neurons are thus emerging as important integrators of environmental and physiological cues within the neuroendocrine reproductive axis, such that they release RFRP-3 peptide to act as a ‘gate’ or ‘brake’ on reproductive function when the afferent inputs they receive suggest conditions are not optimal (see Figure 1). However, there are numerous conflicting data obtained from experiments investigating the roles of RFRP neurons. This review is limited to the neuroendocrine effects of RFRP-3 on GnRH/LH release, primarily in mammals. For a recent review of gonadal paracrine actions of RFRP-3 and GnIH, which may be entirely independent of the neuroendocrine RFRP-3, see [27].

### 2.1. Central RFRP-3 Administration

Many studies have explored the impact(s) of exogenous central RFRP-3 administration on reproductive function. In contrast to the variable results obtained from peripheral administration, these studies mostly support the concept that RFRP-3 acts as a brake on GnRH-induced LH release and reproductive drive. For example, intracerebroventicular (ICV) injection of RFRP-3 to male rats significantly suppressed all facets of male sexual behavior and also reduced plasma levels of LH [28]. ICV RFRP-3 administration in ovariectomized estrogen-primed female rats caused a significant reduction in serum concentrations of LH and FSH [29]. The most obvious inhibitory effects are observed in rodents in response to chronic or acute ICV RFRP-3 infusion during the estradiol-induced preovulatory surge [12,15]. These findings all point to RFRP-3 acting as a central ‘brake’ on the neuroendocrine reproductive axis, perhaps being withdrawn at certain times to promote events such as the preovulatory surge [24]. An interesting exception has been reported in two species of male hamsters and in male mice, where ICV RFRP-3 can stimulate LH secretion [9,12,16]. This effect may be partly due to the interaction of the injected RFRP-3 with the receptor for kisspeptin, Kiss1R [12], to which it has a weak affinity [30].

### 2.2. RFRP Neuronal Activation/Inhibition/Ablation

To further investigate the in vivo role of RFRP-3 on the HPG axis, our lab generated a novel RFRP-Cre mouse line, which we then crossed with Cre-dependent designer receptor (DREADD)- or diphtheria toxin receptor-expressing mice to investigate the effects of endogenous RFRP-3 excitation and inhibition, as well as RFRP neuronal ablation [31]. Chronic RFRP neuronal activation slightly delayed male puberty onset and female reproductive cycle progression, but did not impact adulthood fertility [31]. However, RFRP neuronal excitation was found to reduce LH pulse frequency in adult female mice, but not in males (unpublished, Sawyer IL, Evans MC, Decourt C and Anderson GM). Importantly, female mice exhibiting RFRP neuron ablation or inhibition did not exhibit the stress-induced suppression in pulsatile LH secretion observed in control females, and *Rfrp* gene silencing using targeted shRNA alleviated various stress-induced infertility measures in female rats [32], suggesting that RFRP neurons play a role in mediating the suppressive effects of stress on the neuroendocrine reproductive axis [31] (discussed in greater detail later in this review). Furthermore, ewes treated with a DNA vaccine against *Rfrp*, thereby inhibiting its effect, show elevated gonadotropin levels [33]. These in vivo findings further suggest RFRP-3 functions as an allostatic modulator of the HPG axis.

## 3. The Role of RFRP-3 in Allostatic Reproductive Suppression

In order for RFRP neurons to act as ‘gatekeepers’ of reproductive function, they must be able to receive information relating to both the external and internal environment, which they then integrate and transmit downstream to the GnRH neurons governing fertility. Therefore, information about life cycle stage, time of year, nutritional status, immune health, etc., must be relayed to the RFRP neurons, which in turn must have the appropriate receptors and intracellular signaling pathways to receive and integrate the multitude of inputs before effecting an appropriate downstream response. If conditions are conducive for breeding, the RFRP-3 ‘brake’ on fertility will be removed. However, if conditions are not optimal, RFRP-3 will continue to restrain GnRH release, imposing allostatic reproductive suppression. Here, we will discuss different situations in which RFRP-3 has been proposed to play a role in mediating reproductive suppression.

### 3.1. Pre-Puberty

Puberty is a key developmental milestone, and the timing at which puberty occurs varies greatly across species and also within individuals of the same species. These variations in pubertal timing may be influenced by many factors, including ecological, environmental, and genetic factors. Mechanistically, puberty is characterized by activation of the HPG axis, which is alternatively viewed as a release from inhibition. While the precise mechanisms underpinning puberty onset are still not well understood, RFRP-3 is thought to play a role. Firstly, the RFRP-3 neuropeptide system shows significant changes in activation during the peripubertal period [34], whereby a dramatic decrease in both *Rfrp* expression and *Rfrp*/*c*-*fos* co-expression are observed in the early pre-pubertal stage of female mice. This corroborates other data showing *Rfrp* expression and RFRP cell numbers significantly drops from birth through all postnatal stages in mice [35], and suggests the ‘RFRP-3 brake’ on fertility eases off around puberty. In support of this, the ICV injection of RFRP-3 in prepubertal female rats was shown to delay puberty onset [36], and in prepubertal female mice, RFRP-3 was shown to inhibit LH in an estradiol-dependent manner [37]. Furthermore, data from naked mole rats, a species in which reproduction is limited to a few animals per colony and all others are pubertally suppressed, show that RFRP-3 administration sustains pubertal delay even when animals are removed from the suppressive colony cues [38,39]. RFRP-3 is also implicated in other examples of pubertal delay. For example, glucocorticoid (dexamethasone) treatment during the neonatal period in female mice increases *Rfrp* mRNA, reduces *Gnrh* mRNA and delays pubertal onset [40], suggesting a potential mechanism whereby early life stress may delay puberty via inhibition by RFRP neurons. In contrast to these data, RFRP neuronal activation for 10 days delayed male but not female puberty onset and female reproductive cycle progression [31], and NPFFR1 knockout mice exhibited normal puberty despite being unresponsive to RFRP-3 [41]. However, as is the case with many conditional knockouts, developmental compensation often masks the true physiological role of the targeted deletion.

### 3.2. Reproductive Senescence

Another key reproductive milestone is menopause, or reproductive senescence. While reproductive senescence is often viewed as a result of ovarian insufficiency, ovarian transplants from young animals to old anestrus rats cannot reinitialize estrous cyclicity, whereas young rats can maintain their regular cyclicity after transplantation with old rat ovaries (as reviewed in [42,43]). While age-related changes intrinsic to the GnRH cells and to the efficacy of their secretory output [44,45] contribute to the decline in reproductive competence, corollary evidence supports the possibility that changes in the RFRP-3 neuropeptide system also contribute to reproductive senescence. Firstly, the number of RFRP neurons is downregulated in old age, and there is also a loss of the daily variation in the RFRP neuronal activity in old mice due to changes in their innervation by circadian peptides [46]. The circadian regulation of reproductive function is well established, which we have previously reviewed [47], and RFRP neurons play a role in relaying circadian information to the HPG axis. The increased basal LH release observed in old mice is associated with the decreased number of RFRP neurons [46]; moreover, given the reported inhibitory effect of RFRP-3 on LH secretion in females [48], reduced RFRP-3 ‘braking’ may be part of this loss of gonadotrophic restraint in the aging reproductive axis.

### 3.3. Seasonal Breeding

Seasonal breeding is crucial to the survival and reproductive success of the vast majority of vertebrate species inhabiting temperate regions. Seasonal changes in environmental factors, such as photoperiod, lead to alternating periods of photosensitivity and photorefractoriness in small birds and short-lived mammals to bring about alternating periods of reproductive activity and quiescence, the latter being maintained by an increased potency of negative feedback regulation. In larger long-lived animals, the environmental cues interact with the underlying endogenous rhythm, producing the same reproductive outcome. The timing of reproductive activity, which precedes the birth period by the duration of gestation, ensures that the young are born during a season in which the environmental conditions most favor neonatal survival. Photoperiodic cues can act directly on photoreceptors on the GnRH neuronal network in birds, whereas in mammals they are encoded and communicated to reproductive (and other) brain control centers in the form of elevated secretion of the pineal hormone melatonin during the hours of darkness, which are extended in duration during winter and shorter in summer in temperate regions [49,50].

It would be reasonable to hypothesize that RFRP neuronal activity would be higher during reproductive quiescence than during the breeding season. Consistent with this hypothesis, more RFRP-3 immunoreactive cell bodies were observed during the non-breeding season (summer, long photoperiods) than the breeding season in male macaques (winter, short photoperiods) [51]. Similarly, we observed a significant two-fold increase in the number of RFRP immunoreactive soma in the hypothalamus of female brushtail possums during the summer non-breeding season compared to the breeding season [52]. Similar changes, albeit of a lower magnitude, have been reported in female sheep, another ‘short-day breeder’ [53,54], and RFRP-3 concentration in portal blood is higher in ewes in the summer non-breeding season [18]. However, in small rodents such as Siberian, Syrian, and Turkish hamsters and jerboa, hypothalamic RFRP expression is also increased during long photoperiod exposure [55,56,57,58], despite the fact that these animals are fertile under these conditions in spring and summer. Additionally, the number of RFRP-3 fibers contacting GnRH neurons in both ewes and Siberian hamsters are elevated during long days compared to short days [16,54]. These data are inconsistent with a conserved role for RFRP as a seasonal inhibitor of reproduction across mammalian species; rather, RFRP expression seems to be faithfully tied to photoperiodic length, irrespective of seasonal reproductive status [59]. The situation is different yet again in birds; in long-day breeding quail and house sparrows, GnIH production is increased in response to decreasing photoperiods and treatment with exogenous melatonin [60,61,62]. Thus, while the annual pattern of RFRP-3/GnIH production in sheep is the inverse of that in quail and house sparrows, the times of maximal production are consistent with seasonal reproductive inhibition in these species.

### 3.4. Lactation

There are currently very few studies in which the role of RFRP-3 in lactational infertility has been tested. Conceptually, the output of RFRP neurons might increase to cause lactational amenorrhea since they can inhibit reproductive function. Alternatively, it might decrease since lactation is associated with stress hyporesponsiveness [63] and RFRP-3 stimulates the HPA axis (see following section). In unpublished data from our own lab, we observed that lactating (day 12) rats had about half the amount of *Rfrp* mRNA and immunoreactive RFRP neurons compared to virgin diestrus controls, and this effect was attributable to the presence of elevated circulating prolactin concentrations in the lactating animals (unpublished, Rizwan MZ and Anderson GM). In another study that employed a different experimental design (lactating rats with pups removed were used as controls), the suckling stimulus exerted the opposite effect: it increased *Rfrp* mRNA levels, and the authors suggested this might be a cause of lactational infertility [64]. Clearly, more work is required to further characterize the role, if any, of RFRP-3 in mediating lactational infertility, but the limited current data suggest that prolactin and suckling exert opposite effects on *Rfrp* expression.

### 3.5. Stress

Stress-related reproductive dysfunction is perhaps the most notable form of allostatic reproductive suppression. The reproductive impairments can be subtle, such as reduced hormone secretion, or severe, such as the complete suppression of puberty onset and/or fertility [65]. While many factors contribute to stress-induced reproductive suppression (i.e., metabolic factors, psychosocial factors, immune factors, etc.), RFRP neurons appear to play a central role in integrating and propagating stressor inputs to the neuroendocrine reproductive axis. While different stressors have different physiological ‘fingerprints’, all stressors are able to activate the body’s stress response system—the hypothalamic pituitary adrenal (HPA) axis. The activation of the HPA axis results in the release of glucocorticoids (cortisol in humans and corticosterone in rodents), which act both centrally and peripherally to limit energy-costly processes and achieve allostasis. However, GnRH neurons do not appear to express the glucocorticoid receptor (GR) [66]. Data from our lab and others suggest RFRP neurons play a role in mediating the suppressive effects of HPA axis activation on the HPG axis.

Importantly, approximately half of RFRP neurons express GR [67], and corticosterone administration was shown to increase *Rfrp* gene expression in vitro [68]. Physiologically, both acute and chronic restraint stress have been shown to increase *Rfrp* gene expression in male and female rats [32,67], but this effect was blocked by adrenalectomy [67], highlighting its mediation by glucocorticoids. Furthermore, acute restraint stress was also shown to concomitantly increase RFRP neuronal activation and suppress LH pulsatility in male mice [69]. Data from our lab show that gonad-intact female mice are more prone than males to restraint stress or corticosterone-induced LH pulse suppression. Remarkably, these suppressive effects can be completely overcome by chemogenetic RFRP neuron ablation or inhibition [31]. Similarly, *Rfrp* gene silencing using a targeted shRNA prevented stress-induced infertility in female rats [32]. A single hour of stress exposure in house sparrows increased the number of immunoreactive GnIH neurons [62], indicating a highly conserved role of these neurons in responding to stress. Together, these data suggest a mechanism whereby stress-induced GC release activates RFRP neurons to release RFRP-3, which then puts a brake on GnRH-induced LH release.

#### 3.5.1. Metabolic Stress

Metabolic challenges, such as starvation, inhibit fertility and sexual behaviors. Many of the neurons that provide important input to the reproductive axis are also involved in control of metabolic functions, and this allows the availability of metabolic fuels to be coordinated with fertility [70]. For example, the adipose-derived hormone leptin acts centrally via neurons to permissively modulate the activity of the GnRH neuronal network [71], but this occurs indirectly of GnRH neurons themselves as they do not contain leptin receptors [71]. Since RFRP neurons have been shown to stimulate food intake in mice, rats, sheep, and cynomolgus monkeys [28,72], they could also act as conduits to relay metabolic information to the GnRH neurons and neural circuitry involved in sexual behavior. Indeed, RFRP neurons project to anorexigenic proopiomelanocortin (POMC) cells in mice, and 1 μM RFRP-3 inhibited the firing and hyperpolarized the membrane potential of POMC cells [73]. This effect was able to overcome the excitation of POMC neurons caused by kisspeptin application. The decline in LH secretion in response to a 12-h food restriction was prevented by knockout of NPFFR1 [41]. In contrast, we and others have shown that the percentage of RFRP cells expressing leptin receptor or leptin-induced phosphorylated signal transducer and activator of transcription-3 (STAT3) in both sexes is less than 15% [74,75], and that leptin-deficient mice exhibit either a minor reduction in *Rfrp* mRNA levels [74] or no detectable difference [75] relative to wild-type animals. Furthermore, the postnatal development of RFRP neurons appears to be unaffected by leptin deficiency [74]. Together, these results show that RFRP-3 can modulate metabolic sensing neurons known to influence reproductive function in mice, but are themselves only weakly influenced by metabolic stress.

In Syrian hamsters, the percent of RFRP cells co-expressing c-Fos (a marker of neuronal activation) was significantly increased at 8 and 12 days after food restriction [76]. The effect was speculated to be mediated by orexigenic neuropeptide-Y (NPY) inputs. In this species, therefore, metabolic stress does appear to be able to activate the RFRP neuronal population.

#### 3.5.2. Immune Stress

Immune stress, such as infection and inflammation, can also have a negative impact on fertility [77,78,79]. Experimentally, the bacterial endotoxin lipopolysaccharide (LPS) is used as a model of infection stress because it is known to suppress the steroid-induced preovulatory LH surge and pulsatile LH secretion in female rats. Many factors contribute to the suppressive effects of LPS on the neuroendocrine reproductive axis, including changes to the kisspeptin system, which is critically involved in mediating LPS-induced LH suppression [78]. Interestingly, the RFRP-3 system may also play a role. A septic dose (500 μg/kg i.p.) of LPS in female rats increased both hypothalamic *Rfrp* and *Npffr1* mRNA levels, and these were negatively correlated with *Gnrh* mRNA and serum LH levels [80]. In another study, LPS and RFRP-3 both reduced LH levels in rats [81]. However, the lower dose of LPS used in this study (15 μg/kg) was not found to induce changes in *Rfrp* mRNA despite causing a reduction in serum LH levels [81], suggesting that the involvement of RFRP-3 in immune stress-related reproductive dysfunction depends on its severity. Immune stress can activate the HPA axis and thus impact fertility through GC-induced RFRP-3 release [31,67,69], but perhaps this mechanism of reproductive suppression is only elicited by high doses of LPS.

#### 3.5.3. Psychosocial Stress

As with metabolic and immune stress, which are physiological stressors, psychosocial stress (i.e., perceived stress) can likewise cause allostatic reproductive suppression. However, unlike metabolic and immune stress, which directly threaten an individual’s survival, psychosocial stress is purely a perceived threat. In humans, depression and other mood disorders are examples of psychosocial stress associated with reproductive dysfunction [82]. One common paradigm used to model psychosocial stress in animals is ‘restraint stress’, since the restraint itself is physically harmless but the forced immobilization is perceived as stressful and reliably activates the HPA axis. As mentioned previously, both acute and chronic restraint stress lead to the upregulation of *Rfrp* mRNA, which correlates with a downregulation of serum LH in male rats [67]. GC release was shown to be responsible for the observed increase in *Rfrp* mRNA, since it was prevented by adrenalectomy [67]. We have shown that RFRP neurons are critically involved in the mechanism, whereby HPA activation causes reproductive suppression in female mice, as mice exhibiting RFRP neuronal ablation or silencing (using inactivating DREADDs) do not exhibit the stress-induced suppression in LH pulsatility [31]. Furthermore, central RFRP-3 infusion or activating endogenous RFRP neurons stimulates GC release and anxiety behaviors in mice and rats [30,31,83,84], demonstrating a feedback loop wherein stressful stimuli activate RFRP neurons, which further activate the stress axis and, in turn, RFRP neurons via GC release [31]. This positive feedback loop may help reinforce social dominance hierarchies, such as those observed in nonhuman primates [85] and other eusocial animals like naked mole rats [38]. Not only is elevated *Rfrp* expression associated with suppressed sexual maturation in non-dominant naked mole rats, RFRP-3 administration was sufficient to sustain their suppressed sexual maturation, even when the individuals were removed from the dominance cues [39]. Collectively, these data highlight a critical role for RFRP-3 in the allostatic regulation of reproduction in the context of psychosocial stress.

## 4. Conclusions

The mechanisms underpinning adaptive reproductive suppression are complex and involve a multitude of factors. The RFRP-3 neuropeptide system appears to play a modulatory role in integrating many of these factors and acting as a ‘brake’ on reproductive drive when conditions are unfavorable. RFRP neurons appear to contribute to stress-induced and (in some species) seasonal reproductive suppression, while their role in mediating changes in reproductive competency during different life cycle stages (pre-puberty, lactation and reproductive senescence) is less clear and would benefit from functional data from manipulating RFRP neuronal activity (see Table 1). With the advent of new genetic tools, the precise roles of RFRP neurons in the mechanisms underlying adaptive, and perhaps even maladaptive (e.g., obesity-related infertility), reproductive suppression will hopefully be elucidated and the present conflicting findings clarified.

## Figures and Tables

**Table 1:** A brief summary of some of the key studies investigating the role of the RFRP neuropeptide system in the regulation of adaptive reproduction suppression due to different conditions.

Condition	Evidence for RFRP-3 Involvement	Animal Model	Type of Evidence	References
Pre-puberty	RFRP-3 cell number, *Rfrp* expression, and *Rfrp + cFos* co-expression decrease during early pubertal transition	Female mice	Correlational	[34,35]
	ICV injection of RFRP-3 delays puberty onset	Female rats	Functional	[36]
	RFRP-3 administration marginally sustains social subordination-induced pubertal delay	Male and female naked mole rats	Functional	[39]
	NPFFR1 (RFRP-3 receptor) knockout has no effect on puberty	Male and female mice	Inconclusive	[41]
	RFRP neuronal activation delays puberty onset	Male mice (no effect in females)	Functional	[31]
Reproductive Senescence	RFRP-3 cell numbers are further downregulated	Female mice	Correlational	[46]
	Loss of the daily variation in the RFRP neuronal activity	Female mice	Correlational	[44]
Seasonal Breeding	Increased RFRP cells in non-breeding season	Female brushtail possums	Correlational	[52]
	Increased RFRP cells in non-breeding season	Male rhesus macaques	Correlational	[51]
	Increased RFRP cells in non-breeding season	Female sheep	Correlational	[53,54]
	Increased RFRP-3 in portal blood in non-breeding season	Female sheep	Correlational	[18]
	Increased RFRP-3 cells in breeding season	Male and female hamsters	Contradictory	[55,56,57,58]
Lactation	Suckling stimulus increases *Rfrp* expression	Female rats	Correlational	[64]
Psychosocial Stress	Acute and chronic restraint stress increase *Rfrp* expression, which is prevented by adrenalectomy	Male rats	Correlational	[67]
	Knockdown of hypothalamic RFRP-3 prevents chronic stress-induced embryo resorption	Female rats	Functional	[32]
	Acute restraint stress increased RFRP neuronal activation and concomitantly decreased LH pulsatility	Male mice	Correlational	[69]
	RFRP neuronal silencing or ablation prevents acute restraint stress-induced suppression in LH pulsatility	Female mice (no effect in males)	Functional	[31]
Metabolic Stress	ICV RFRP-3 administration inhibits POMC neurons	Male and female mice	Correlational	[73]
	NPFFR1 (RFRP-3 receptor) knockout prevents decline in LH pulses in response to 12 h food restriction	Male mice	Functional	[41]
	Leptin deficiency does not affect postnatal development of RFRP neuropeptide system	Male and female mice	Inconclusive	[74]
	RFRP-cFos co-expression is increased 8 and 12 days after food deprivation	Female Syrian hamsters	Correlational	[76]
Immune Stress	A septic dose of LPS decreased serum LH and increased both hypothalamic *Rfrp* and *Npffr1* expression	Female rats	Correlational	[80]
	A low dose of LPS decreased serum LH but had no effect on RFRP neuropeptide system	Female rats	Inconclusive	[80]

## Data Availability

No new data were created or analyzed in this study. Data sharing is not applicable to this article.
